# Proteomic analysis of heat-stable proteins revealed an increased proportion of proteins with compositionally biased regions

**DOI:** 10.1038/s41598-022-08044-z

**Published:** 2022-03-14

**Authors:** Hongsun Park, Tomoyuki Yamanaka, Nobuyuki Nukina

**Affiliations:** 1 Laboratory of Structural Neuropathology, Doshisha University Graduate School of Brain Science, 1-3 Miyakodanitatara, Kyotanabe-shi, Kyoto, 610-0394 Japan; 2 Department of Neuroscience of Disease, Brain Research Institute, Niigata University, Niigata, Japan

**Keywords:** Protein aggregation, Neurodegenerative diseases

## Abstract

Intrinsically disordered proteins (IDPs) have been in the spotlight for their unique properties, such as their lack of secondary structures and low sequence complexity. Alpha-synuclein and tau are representative disease-related IDPs with low complexity regions in their sequences, accumulating in the brains of patients with Parkinson disease and Alzheimer disease, respectively. Their heat resistance in particular was what attracted our attention. We assumed that there exist many other unidentified proteins that are resistant to heat-treatment, referred to as heat-stable proteins, which would also have low sequence complexity. In this study, we performed proteomic analysis of heat-stable proteins of mouse brains and found that proteins with compositionally biased regions are abundant in the heat-stable proteins. The proteins related to neurodegeneration are known to undergo different types of post-translational modifications (PTMs) such as phosphorylation and ubiquitination. We then investigated the heat-stability and aggregation properties of phosphorylated synuclein and tau with different phosphorylation sites. We suggest that PTMs can be important factors that determine the heat-stability and aggregation properties of a protein. IDPs identified in the heat-stable proteins of mouse brains would be candidates for the pathogenic proteins for neurodegeneration.

## Introduction

Many neurodegenerative diseases are characterized by the aggregation of misfolded proteins such as alpha-synuclein (⍺-syn) in Parkinson disease (PD) or tau in Alzheimer disease (AD)^1,2^. These disease-related proteins are known as intrinsically disordered proteins (IDPs), as they lack well-defined three-dimensional structures. Recently, the importance of characterizing these IDPs, also known as intrinsically unstructured proteins^3^ or natively denatured proteins^4^, has become widely appreciated.

IDPs are characterized by the presence of intrinsically disordered regions (IDRs), as well as by their low proportions of bulky hydrophobic amino acids and high proportions of charged and hydrophilic amino acids^5^. Unlike folded domains, IDRs do not adopt stable secondary or tertiary structures, and a subset of disordered protein regions, termed low complexity regions (LCRs), exhibit compositional bias towards a small set of amino acids^6^. It is assumed that long (> 30 residues) disordered segments are found to occur in 33% of eukaryotic proteins, and 22% of human disease mutations occur in IDRs^7^, which are frequently associated with various human diseases such as genetic diseases, diabetes and neurodegenerative diseases^8,9^. These proteins with IDRs can undergo different types of PTMs such as phosphorylation, ubiquitination and acetylation, which can lead to pathological situations due to the modulation of their normal functions^10,11^.

The aggregation of ⍺-syn and tau can also be affected by diverse PTMs. In particular, S129 phosphorylation in ⍺-syn has attracted the most attention since approximately 90% of ⍺-syn deposited in Lewy bodies (LBs) are selectively phosphorylated at this site^12^. This phosphorylated synuclein is also observed in other synucleinopathies, including multiple system atrophy, Hallervorden-Spatz disease, pure autonomic failure and LB variant of AD (LBVAD), suggesting a close relationship between S129 phosphorylation in ⍺-syn and its aggregation^13^. Phosphorylation of tau was also shown to be involved in protein aggregation and cytotoxicity^14,15^. Early studies have demonstrated immunochemically and biochemically that tau is a component of paired-helical filaments (PHFs) forming the neurofibrillary tangles (NFTs) in AD brains and was assumed to contribute to pathological processes in AD when highly phosphorylated^16–20^. Enhanced immunoreactivity of tau deposition in AD tissues was observed with the phosphorylation-dependent antibodies AT8 (epitope S199/S202/T205), PHF-1 (epitope S396/S404) and phospho-tau (epitope S262)^21,22^. Approximately 45 phosphorylation sites were found in insoluble aggregated tau purified from AD brains, while approximately 10 phosphorylation sites were detected in soluble tau extracted from normal brains. In addition, at least 16 phosphorylation sites have been found in brains of progressive supranuclear palsy (PSP), another tauopathy^15^. The S/T phosphorylation of tau is the most studied modification that can affect a total of 79 residues in full-length tau of 441 residues in the central nervous system^23^. In AD, at least 30 S/T residues are phosphorylated by several kinases such as GSK-3, cdk5, p38, MARK or CKII^24^. Among these kinases, it has been established that GSK-3 is implicated in the formation of aberrant tau aggregates in certain mouse models^25,26^.

In addition to these features of ⍺-syn and tau, they are known as heat-resistant^27,28^. Therefore, we hypothesized that there are still many other unidentified heat-stable proteins (i.e. thermo-resistant or heat-resistant proteins) that would also contain LCRs in their sequences. To address this issue, we purified and analyzed heat-stable proteins from mouse brains with liquid-chromatography mass spectrometry (LC–MS/MS) and investigated their properties using analytic software for proteome. Finally, we suggest that phosphorylation of the heat-stable proteins may affect the heat-stability and contribute to the aggregation of proteins.

## Results

### Heat-stable proteins were purified from mouse brains

**Figure 1:**
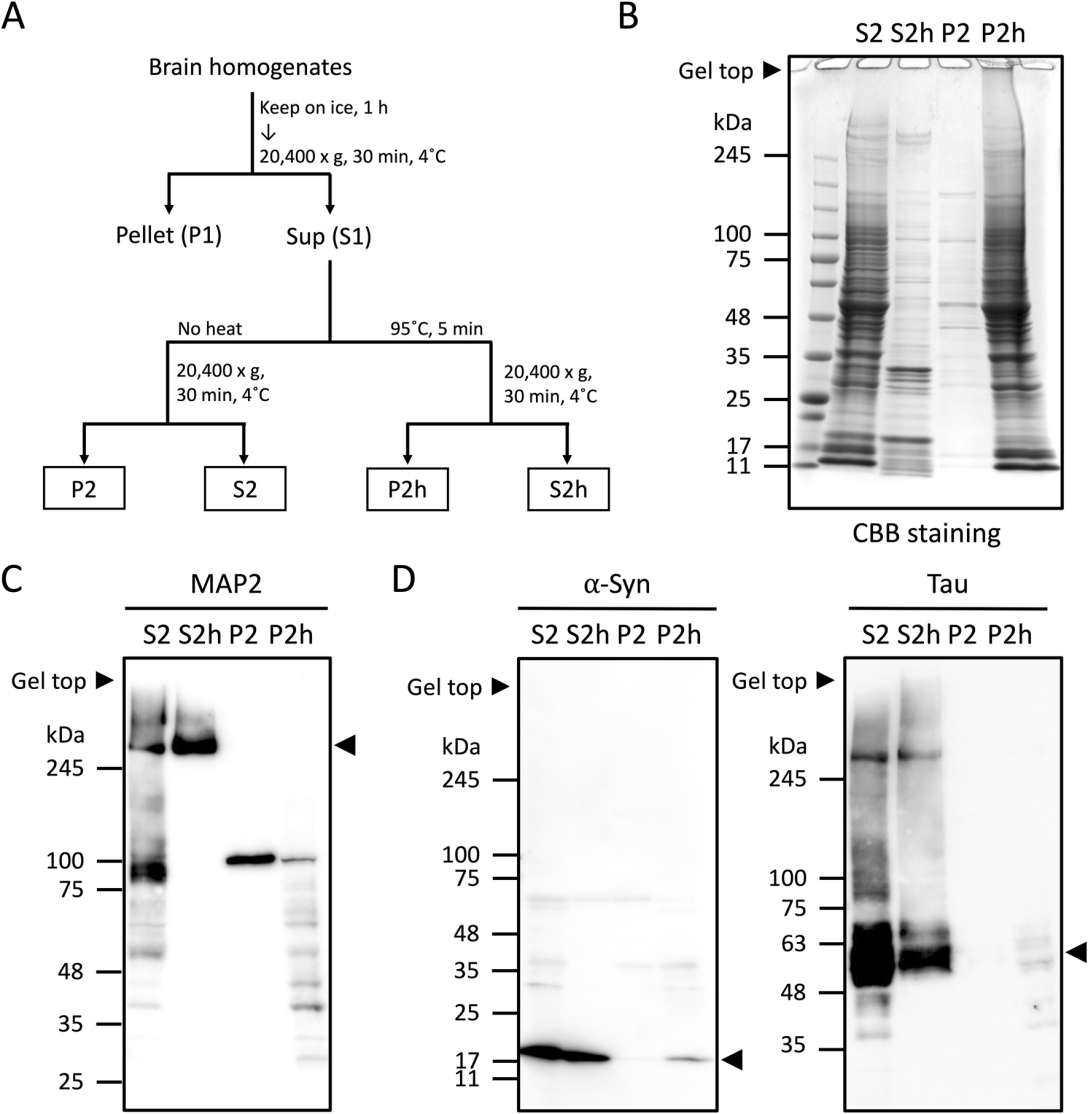
Purification of heat-stable proteins. **(A)** Mouse brains were subjected to subcellular fractionation. (**B)** Equal amounts of fractions were subjected to SDS-PAGE and stained with CBB. (**C)** Western blot of each fraction stained with anti-MAP2 antibody. **(D)** Western blot of each fraction stained with anti-⍺-syn and anti-tau antibodies. Arrowheads indicate the representative bands of each protein.

The S1 fraction of 22-week-old C57BL6J mouse brains were obtained by subcellular fractionation based on the preparation of microtubule associated proteins^17^. The fractions were incubated at 95 °C for 5 min and centrifuged at 20,400×g for 30 min at 4 °C. The supernatant and the pellet after the heat-treatment were denoted as S2h and P2h respectively, and non-heat-treated P2 and S2 fractions were also prepared as controls (Fig. 1A). While the total amount of proteins in S2h were significantly reduced (Fig. 1B), we confirmed that MAP2, a known heat-stable protein, and ⍺-syn and tau were detected in the S2h heat-stable fraction after the heat-treatment (Fig. 1C,D). After the heat-treatment, most of ⍺-syn and tau remained in the S2h fraction, although a small amount of ⍺-syn and tau were detected in P2h (Fig. 1D). The CBB-stained bands observed in the S2 and P2h fractions look similar (Fig. 1B), indicating that most proteins are not heat-stable. Therefore, heat-stable proteins in the S2h fractions may have distinct features compared to other fractions.

### Heat-stable proteins contained low complexity regions

**Figure 2:**
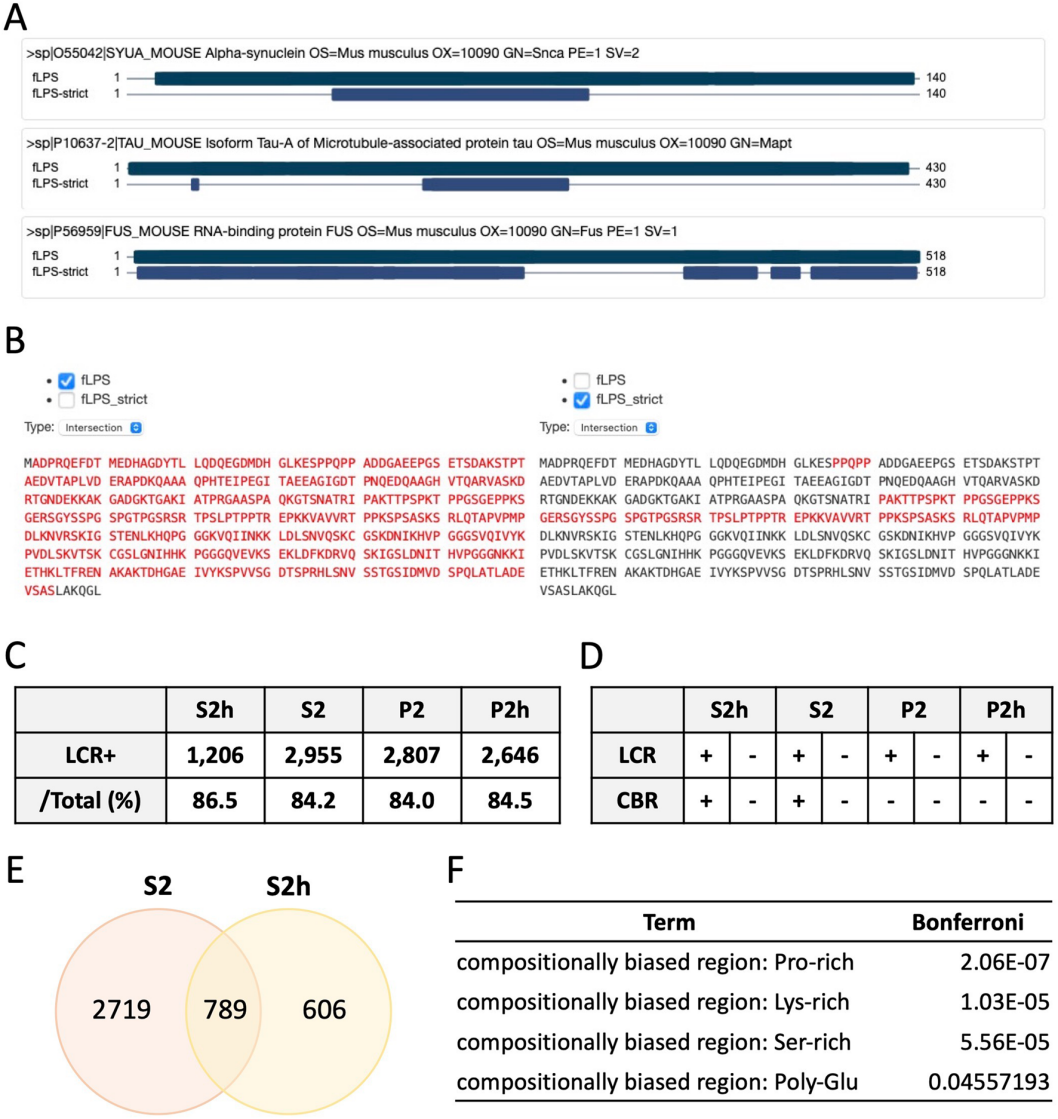
Analysis of LCRs in protein sequences using PlaToLoCo and DAVID. **(A)** PlaToLoCo analysis showed that ⍺-syn, tau and FUS contained LCRs in their sequences. **(B)** The sequences of mouse tau were analyzed by PlaToLoCo. The regions marked by red indicate LCRs. fLPS describes most parts of the sequence of tau as LCRs, while fLPS-strict does more limited detection of LCRs. **(C)** The number of proteins containing LCRs in each fraction. **(D)** The presence or absence of LCRs and CBRs in each fraction. **(E)** The number of proteins in the S2 and S2h fractions. 789 proteins were shared in common between S2 and S2h. **(F)** The sequence features of the S2h fraction analyzed by DAVID. The proteins shared between S2 and S2h were excluded.

Since ⍺-syn and tau were detected in S2h, we assumed there were many other heat-stable proteins in this fraction. To identify known and unknown heat-stable proteins, each fraction was digested by trypsin and then subjected to LC–MS/MS. 3508 proteins in S2, 1395 proteins in S2h, 3343 proteins in P2 and 3132 proteins in P2h were identified (Supplementary information 1 and 2). Then, we investigated whether the proteins in each fraction contained LCRs in their sequences using PlaToLoCo, PLAtform of TOols for LOw COmplexity^29^. PlaToLoCo is the first web meta-server for visualization and annotation of LCRs in proteins, integrating and collecting the output of five different state-of-the-art tools for the discovery of LCRs. Among five tools they provided including SEG or CAST, we used fLPS. Built on the previous LPS algorithm, fLPS is developed for the fast discovery of compositional biases in proteins, using a number of new measures, which substantially increase efficiency. This tool outputs all single-residue and multiple-residue LPSs (low-probability subsequences), along with the results of a simple calculation of compositional biases over the whole protein sequence^30^. With this tool, we confirmed that some known proteins containing LCRs such as ⍺-syn, tau and FUS (fused in sarcoma) had LCRs in their sequences (Fig. 2A). However, fLPS describes most parts of the sequence of tau as LCRs, while fLPS-strict, the predefined fLPS tool, does more limited detection (Fig. 2B). Therefore, we decided to use fLPS-strict to identify proteins containing at least one LCR. Among 1395 proteins identified in the S2h fraction, 1206 proteins (86.5%) contained LCRs, and 189 proteins did not (Supplementary information 1). For the S2, P2 and P2h fractions, 2955 (84.2%), 2807 (84.0%) and 2646 (84.5%) proteins were found to contain at least one LCR (Fig. 2C). Although PlaToLoCo is an easy and useful tool for investigating LCRs in protein sequences, it was difficult to distinguish the sequence features of each fraction, since most proteins identified in the non-heat-treated and insoluble fractions also contained at least one LCR in their sequences. Therefore, we decided to analyze the sequence features of the identified proteins in each fraction by DAVID Bioinformatics Resources 6.8 (DAVID).

### Proteins with compositionally biased regions were found abundantly in the heat-stable fraction

DAVID provides a comprehensive set of functional annotation tools for investigators to understand the biological meaning behind large lists of genes. For the proteins in the S2 fraction, the top 10 sequence features included mitochondrion transit peptide, nucleotide phosphate-binding regions of GTP, ATP and NADP, substrate- and metal ion- binding sites (Magnesium), effector region, lipid moiety-binding region (S-geranylgeranyl cysteine), PCI domain and WD 5 repeat (Table 1). Since no specific amino acid rich proteins were described as abundant in this fraction, biased amino acid rich proteins may not be the majority. On the other hand, the proteins in the S2h fraction, which are resistant to heat, had specific amino acid rich sequences. The top 10 sequence features of heat-stable proteins included Pro-rich, Lys-rich, Glu-rich, Ser-rich and Arg/Ser-rich (Table 2). These features suggest that many heat-stable proteins contain biased amino acid rich sequences. As expected, the proteins in the P2 and P2h fractions were not as rich in specific amino acids as those in S2h (Table S1 and S2). Also, we investigated whether the presence or absence of LCRs has any relationship with the existence of compositionally biased regions (CBRs) in protein sequences (Fig. 2D). As a result, with the presence of LCRs, the heat-stable proteins in S2h included many CBRs such as Pro-rich, Glu-rich, Lys-rich, Ser-rich, Arg/Ser-rich, Poly-Glu and Gly-rich (Table 3), while the S2 proteins contained only one CBR, Glu-rich (Table S3). There was about a tenfold increase in the proportion of proteins containing CBRs in S2h (29.2%) compared to S2 (2.6%) (Table S4, Supplementary information 1). The proteins identified in the P2 and P2h fractions were not abundant with CBRs, regardless of whether or not they contained LCRs in their sequences (Tables S5, S6). Since S2h proteins and S2 proteins shared 789 proteins in common (Fig. 2E, Supplementary information 1), DAVID analysis was performed again with the proteins in S2h after the proteins shared between S2 and S2h were excluded. We found that heat-stable proteins with CBRs such as Pro-rich, Lys-rich, Ser-rich and Poly-Glu regions in their sequences were the majority in the S2h fraction (Fig. 2F). In addition, the sequence features of the 789 proteins that S2 and S2h shared in common included Glu-rich, a CBR (Table S7), while no CBR was found in S2 without the shared proteins between S2 and S2h (Table S8). Taken together, proteins with CBRs were found abundantly in the heat-stable proteins, and we suggest that combining these two methods of bioinformatic analysis could be useful for investigating the sequence features of proteins containing LCRs.

**Table 1:** The sequence features of S2 proteins analyzed by DAVID.

	Term	Count	%	List Total	Bonferroni
1	Transit peptide: Mitochondrion	201	5.7	3395	8.74E−26
2	Nucleotide phosphate-binding region: GTP	129	3.7	3395	2.28E−15
3	Nucleotide phosphate-binding region: ATP	285	8.1	3395	1.59E−12
4	Binding site: Substrate	110	3.1	3395	1.03E−08
5	Metal ion-binding site: Magnesium	54	1.5	3395	2.92E−08
6	Short sequence motif: Effector region	48	1.4	3395	9.37E−07
7	Lipid moiety-binding region: S-geranylgeranyl cysteine	51	1.5	3395	1.09E−06
8	Domain:PCI	16	0.5	3395	1.67E−04
9	Repeat:WD 5	73	2.1	3395	6.39E−04
10	Nucleotide phosphate-binding region: NADP	31	0.9	3395	0.0014012
11	Repeat: HEAT 3	24	0.7	3395	0.00150928
12	Repeat: WD 3	80	2.3	3395	0.00158049
13	Domain: SH3	60	1.7	3395	0.00188571
14	Active site: Proton acceptor	188	5.4	3395	0.00254292
15	Repeat: WD 4	76	2.2	3395	0.00277027
16	Repeat: WD 2	80	2.3	3395	0.00400307
17	Repeat: WD 1	80	2.3	3395	0.00400307
18	Repeat: HEAT 4	22	0.6	3395	0.0041559
19	Repeat: WD 6	59	1.7	3395	0.00570746
20	Binding site: NADP	17	0.5	3395	0.00624824
21	Repeat: HEAT 5	19	0.5	3395	0.01006313
22	Repeat: HEAT 1	25	0.7	3395	0.01214636
23	Repeat: HEAT 2	25	0.7	3395	0.01214636
24	Region of interest: Calmodulin-binding	25	0.7	3395	0.01214636
25	Calcium-binding region: 1	47	1.3	3395	0.01612155
26	Region of interest: Substrate binding	50	1.4	3395	0.01925011
27	Domain: Guanylate kinase-like	15	0.4	3395	0.03367096
28	Splice variant	988	28.2	3395	0.03500524

**Table 2:** The sequence features of S2h proteins analyzed by DAVID.

	Term	Count	%	List Total	Bonferroni
1	Compositionally biased region: Pro-rich	105	7.5	1340	2.73E−07
2	Compositionally biased region: Lys-rich	29	2.1	1340	1.21E−04
3	Compositionally biased region: Glu-rich	47	3.4	1340	1.34E−04
4	Domain: EF-hand 1	36	2.6	1340	1.83E−04
5	Domain: EF-hand 2	35	2.5	1340	5.11E−04
6	Repeat: 8	19	1.4	1340	9.17E−04
7	Compositionally biased region: Ser-rich	59	4.2	1340	0.00104652
8	Calcium-binding region: 1	28	2	1340	0.00161438
9	Compositionally biased region: Arg/Ser-rich (RS domain)	11	0.8	1340	0.00291166
10	Domain: WH2	10	0.7	1340	0.00626676
11	Repeat: 7	19	1.4	1340	0.00848903
12	Domain: EF-hand 3	22	1.6	1340	0.00851955
13	Repeat: 4	27	1.9	1340	0.00888128
14	Calcium-binding region: 2	25	1.8	1340	0.00959587
15	Domain: RRM	25	1.8	1340	0.01321439
16	Splice variant	420	30.1	1340	0.01581153
17	Repeat: 9	15	1.1	1340	0.02925218
18	Repeat: 10	15	1.1	1340	0.02925218
19	Repeat: 11	14	1	1340	0.03091004

**Table 3:** The sequence features of S2h proteins, with and without LCRs, analyzed by DAVID.

S2h	
LCR+	LCR−
1206 (86.5%)	189
Compositionally biased region: Pro-rich	Binding site: Substrate
Splice variant	Domain: EF-hand 1
Compositionally biased region: Glu-rich	Calcium-binding region: 3
Compositionally biased region: Lys-rich	Site: Interaction with phosphoserine on interacting protein
Compositionally biased region: Ser-rich	Domain: EF-hand 2
Repeat: 8	Calcium-binding region: 4
Repeat: 4	Domain: EF-hand 4
Compositionally biased region: Arg/Ser-rich (RS domain)	Domain: EF-hand 3
Domain: RRM	Calcium-binding region: 1
Repeat: 7	
Domain: WH2	
Repeat: 10	
Repeat: 9	
Compositionally biased region: Poly-Glu	
Repeat: 11	
Repeat: 6	
Repeat: 2	
Compositionally biased region: Gly-rich	

See Supplementary information 1 for each protein list.

### The heat-stability and aggregation properties of phosphorylated synuclein and tau were affected by heat-treatment

**Figure 3:**
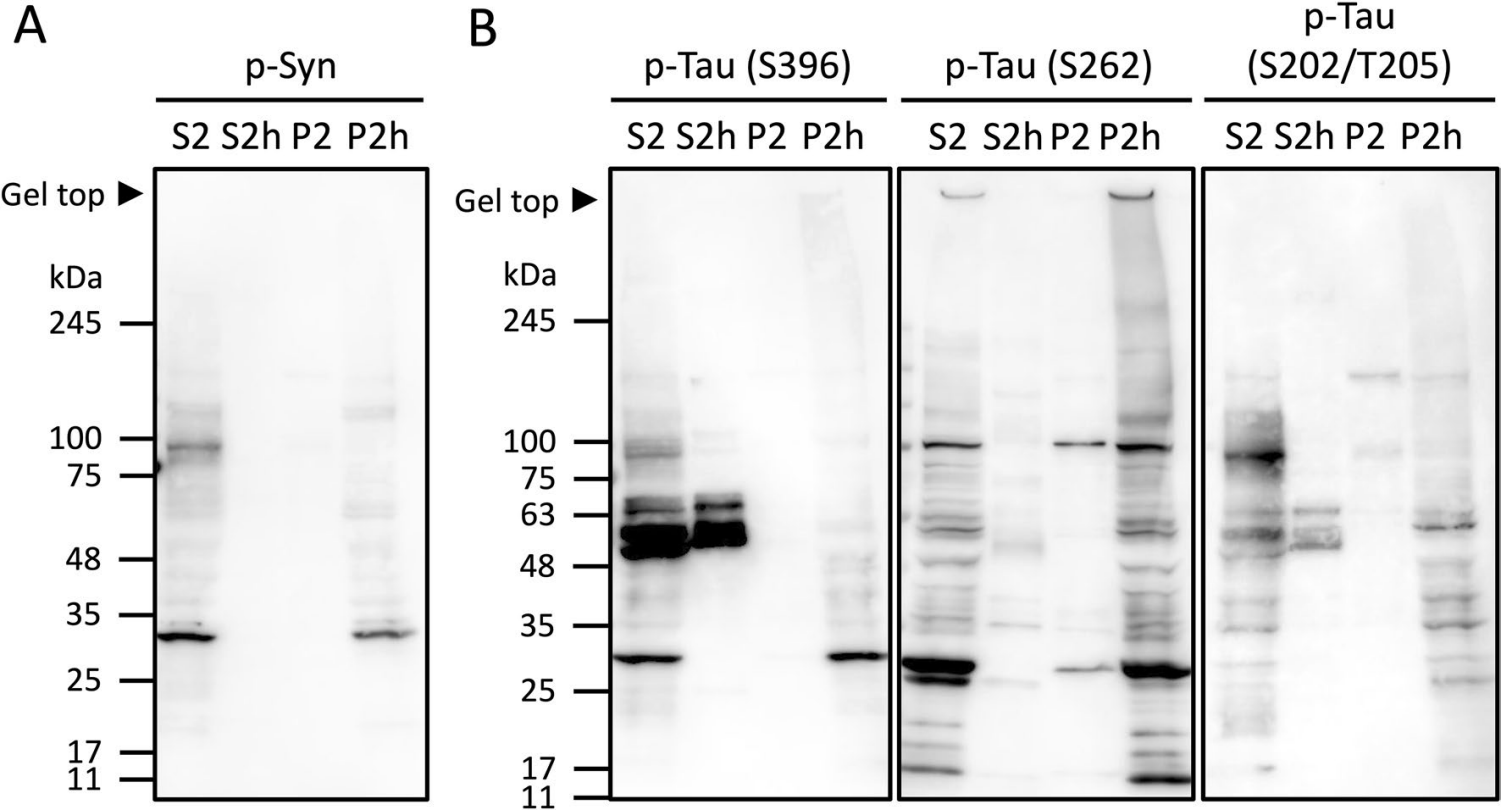
Western blot analysis of phosphorylated proteins. Western blot of each fraction stained with the antibodies to p-syn **(A)**, p-tau S396, p-tau S262 and p-tau S202/T205 (AT8) **(B)**.

Since phosphorylation is the most common PTM that could contribute to protein aggregation, we investigated whether phosphorylated synuclein (p-syn) and tau (p-tau) remained in the heat-stable fractions. By Western blot, we found that p-syn was detected in non-heat-treated S2 fraction but not in heat-treated S2h fraction, indicating that p-syn is less heat-stable compared to ⍺-syn and accelerated to form aggregates by the heat-treatment (Fig. 3A).

For p-tau, its heat-stability and ability to form aggregates depended on the phosphorylation sites (Fig. 3B). Tau phosphorylated at S396 residue (p-tau S396, hereafter the residue numbers are corresponding to the number of 441-residue human tau) was the most heat-stable compared to p-tau S262 or S202/T205 (AT8). On the other hand, p-tau S262 tends to form large sized aggregates including those in the gel top compared to others. Taken together, the heat-stability and protein aggregation could be affected by the phosphorylation of proteins depending on the phosphorylation sites.

### The heat-stability and aggregation properties of FUS and TDP-43 were affected by heat-treatment

**Figure 4:**
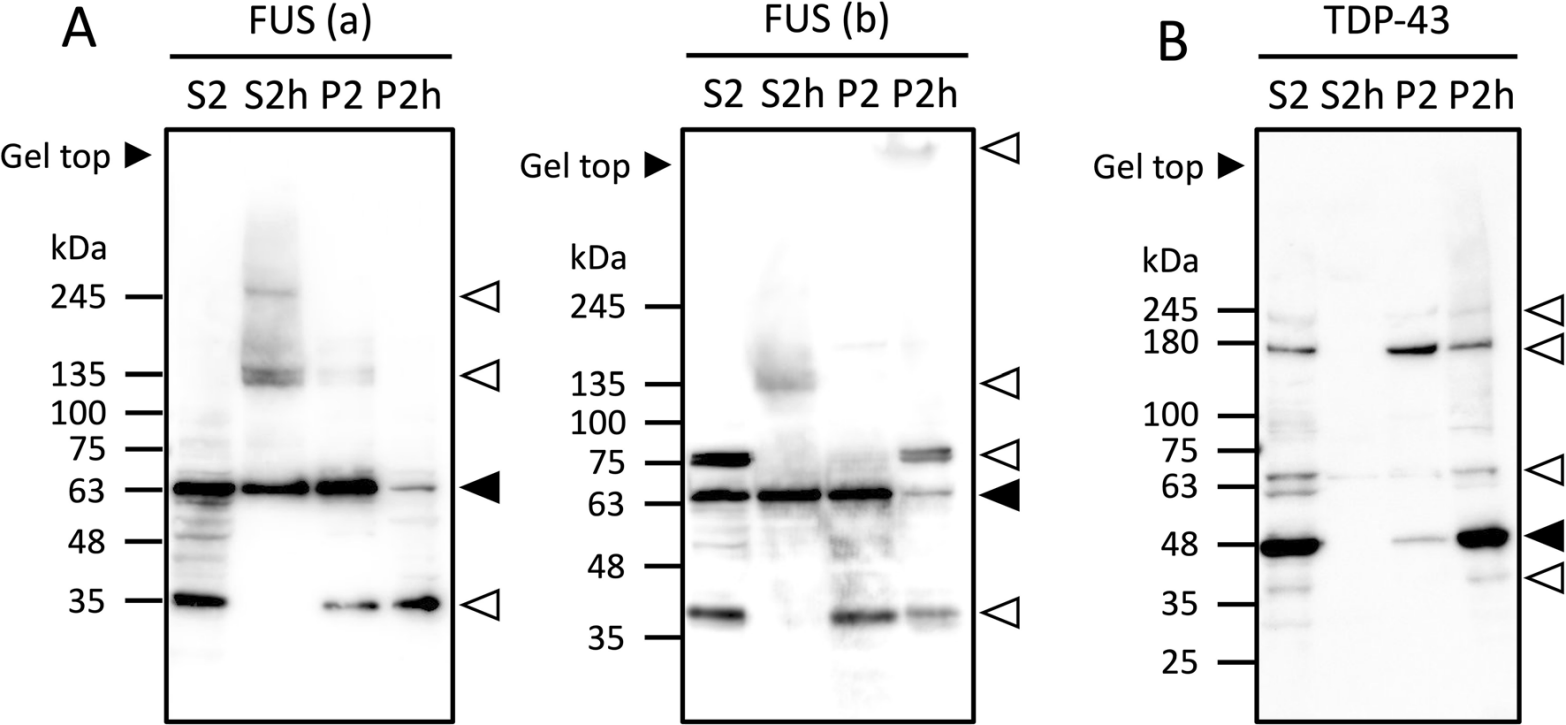
Western blot analysis of FUS and TDP-43. **(A)** Western blot of each fraction stained with anti-FUS antibodies (a: Proteintech, 11570-1-AP; b: Sigma-Aldrich, HPA008784). **(B)** Western blot of each fraction stained with anti-TDP-43 antibodies. Black arrowheads are the representative bands of each protein. White arrowheads indicate large sized isoforms and small sized fragments of each protein.

We further investigated the heat-stability and aggregation properties of other disease proteins containing LCRs, such as FUS and TDP-43 (TAR DNA-binding protein 43). FUS (also designated TLS), the mutation of which causes amyotrophic lateral sclerosis (ALS), was identified in S2h as well as in the other fractions by LC–MS/MS (Supplementary information 1 and 2). This result corresponded to the Western blot result as the bands of FUS were detected in all the fractions (Fig. 4A). As a strong band of the main isoform (65 kDa) was detected in S2h, we considered FUS as a heat-stable protein. On the other hand, the bands with smaller or larger sizes (35 kDa, 75 kDa and the gel top bands) compared to this band was detected in P2h, suggesting that FUS aggregates are composed of the different isoforms or fragments with various sizes rather than only the main isoform, and the bands around 135 kDa and 245 kDa in S2h could be oligomers of FUS. Previously, we found that the N-terminal LCR of FUS/TLS (Translocated in Liposarcoma) mediates co-aggregation with mutant huntingtin (Htt)^31^ and later identified FUS/TLS as a tightly bound component of the Htt aggregates in vivo using Htt aggregates isolated from Huntington disease model mice^32^. Since the polyglutamine tract itself is an LCR, our study suggested deleterious consequences of the interaction between FUS/TLS and polyglutamine through the LCRs. Taken together, the heat-stability of FUS might change depending on the size of fragments, and the presence of LCRs might be closely implicated in the heat-stability and aggregation properties of FUS.

TDP-43, another pathogenic protein of ALS, did not show any representative bands in S2h (Fig. 4B), suggesting that this protein is not a heat-stable protein. We also confirmed that TDP-43 was not identified in S2h by LC–MS/MS, which corresponds with the Western blot result (Supplementary information 1). The aggregation properties of TDP-43 was clear since the main isoform (43 kDa) and the isoforms or fragments with various sizes (40 kDa, 65 kDa, 180 kDa and 245 kDa) were all detected as strong bands in P2h. This suggests that the heat-treatment affected the aggregation properties of TDP-43.

## Discussion

Generally, the cores of globular proteins are rich in hydrophobic amino acids that stabilize the protein structures, while the surfaces of globular proteins are rich in polar and charged amino acids that interact favorably with water. On the other hand, IDPs contain a low quantity of hydrophobic amino acids but are rich in polar and charged amino acids with low sequence complexity^33,34^. LCRs or CBRs were found in many disease-related IDPs such as ⍺-syn and tau. Focusing on the fact that ⍺-syn and tau were resistant to heat, we hypothesized and confirmed that there are many other heat-stable proteins that contain LCRs, raising the possibility that those proteins have various implications for human diseases.

Neurodegenerative diseases such as PD, AD and ALS are characterized by the progressive degeneration of neurons in the central nervous system. From a molecular perspective, the hallmark shared by these neurodegenerative disorders is the misfolding and aberrant aggregation of the disease proteins. This feature is also characteristic of prion disease and other amyloidosis inside and outside the central nervous system, suggesting that neurodegenerative disorders are part of a much larger family of protein misfolding disorders^13,35^.

Although ⍺-syn and tau are heat-stable proteins, a low quantity of the proteins were also detected in the insoluble fractions after the heat-treatment (Fig. 1D). We assumed that those detected in the insoluble fractions are p-syn and p-tau and confirmed that both p-syn and p-tau existed in each fraction by Western blot (Fig. 3). We further investigated whether phosphorylation or any other PTMs of these disease proteins are implicated in their heat-stability and aggregation properties. Since p-syn positive bands were detected in S2 and P2h by Western blot, we assumed that the S129 phosphorylated p-syn peptide would be identified in the S2 and P2h fractions by LC–MS/MS. However, the peptides including S129, with or without phosphorylation, were not identified in any fraction by LC–MS/MS. This might be due to the fact that the peptide including S129 became insoluble, making it difficult to be analyzed by LC–MS/MS. Aside from phosphorylation, carbamylated peptides were identified in the S2h and S2 fractions, five peptides in S2h and one peptide in S2. This might suggest that the heat-stability of ⍺-syn is affected by carbamylation. However, carbamylation of lysine residues and protein N-termini, which is a hallmark of aging, can be artificially introduced during sample preparation with urea^36^. Therefore, the direct effect of carbamylation on the properties of ⍺-syn remains to be unclear. Based on the result of Western blot, we suppose S129 phosphorylation affects the heat-stability and enhances the aggregation properties of ⍺-syn.

**Figure 5:**
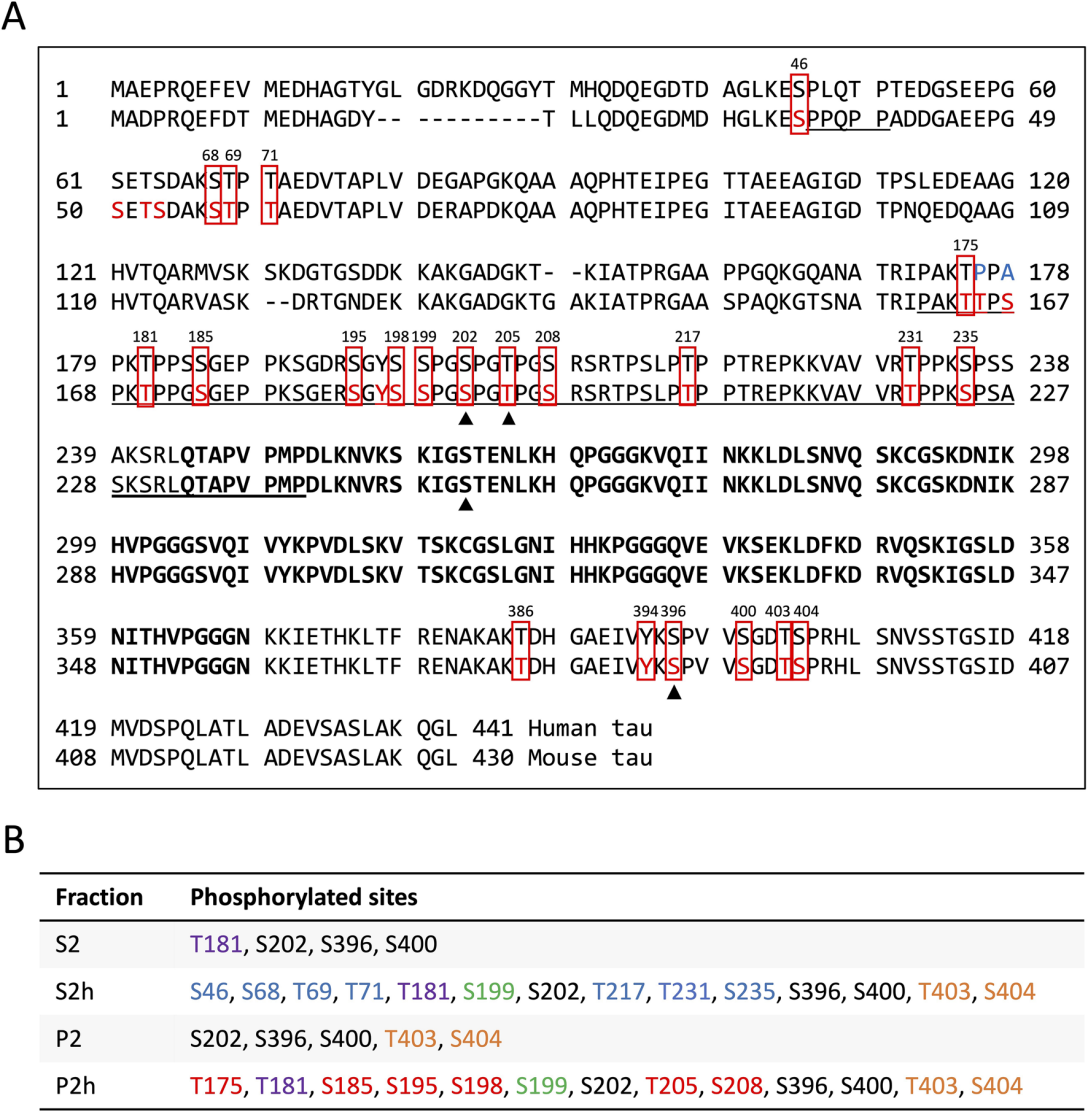
Tau phosphorylation sites identified in this study. **(A)** Alignment of 441-residue human tau and 430-residue mouse tau sequences with identified phosphorylation sites. Phosphorylated sites of mouse tau detected by LC–MS/MS in this study are marked in red. The red boxes indicate conserved phosphorylation sites in human tau. Nonconserved phosphorylation sites in human is marked in blue. Arrowheads indicate the phosphorylation sites we tested by Western blot. Bolded letters show the repeat domains (244–368 aa). Underlined regions indicate the LCRs analyzed by PlaToLoCo. **(B)** Phosphorylated sites of tau identified in each fraction. The phosphorylated sites specifically identified in S2h and P2h are marked in blue and red, respectively. The phosphorylated sites shared in all fractions are marked in black, and those shared between S2, S2h and P2h are marked in purple. The phosphorylated sites shared between S2h and P2h are marked in green, and those shared between S2h, P2 and P2h are marked in orange.

The heat-stability and aggregation properties of p-tau differed by its phosphorylation sites we tested by Western blot (S396, S262 and S202/T205) (Fig. 3B). We assumed that the tau peptides including those phosphorylation sites would be identified by LC–MS/MS and found some mouse tau peptides phosphorylated at several phosphorylation sites including S396 and S202/T205. We then compared the sequences of 430-residue mouse tau (UniProt: P10637-2) with 441-residue human tau (UniProt: P10636-8). Most of the identified phosphorylation sites of mouse tau corresponded to human tau phosphorylation sites (Fig. 5A). P-tau S262 was not identified in any fractions, but tau peptides containing S262 residue (KIGSTENLKH, 248–257 aa) without phosphorylation were identified in the S2h and P2h fractions. Since S262 residue could be analyzed by MS/MS analyses of tau prepared from various tauopathies^37^, the reason that p-tau S262 was not identified in this study might be that the amount of p-tau S262 peptides was not enough to be analyzed by LC–MS/MS. However, we could detect p-tau S262 by Western blot and found that p-tau S262 formed more aggregates compared to p-tau S396 or p-tau S202/T205 regardless of the size of isoforms or fragments. This suggests that the insolubility and/or low amount of p-tau peptides within the core of tau aggregation makes it difficult to be analyzed by LC–MS/MS. In fact, no phosphorylated sites of mouse tau within the aggregation core region, i.e. repeat domain, were identified (Fig. 5A).

Aside from S262, many phosphorylated sites were identified in each fraction (Fig. 5B). In S2 and S2h fractions, 4 phosphorylated sites and 14 phosphorylated sites including S396 were identified, respectively. As p-tau S396 showed strong bands (48–63 kDa) both in S2 and S2h by Western blot, S396 might not affect the heat-stability of tau. 7 phosphorylated sites (S46, S68, T69, T71, T217, T231 and S235) specifically identified in S2h either might not affect the heat-stability of tau, or those phosphorylation could increase heat-stability.

In the P2h fraction, 13 phosphorylated sites including S396 and S202/T205 were identified. Since S396 and S202/T205 were also identified in the heat-treated S2h fraction, those phosphorylation sites might not be the specific sites that are implicated in tau aggregation. However, 30 kDa small fragments of p-tau S396 and S202/T205 showed strong bands in S2 and P2h but not in S2h or P2 (Fig. 3B), suggesting that small sized tau fragments containing those phosphorylation sites are less resistant to heat and tend to form more aggregates after the heat-treatment compared to large isoforms. In P2h, 6 phosphorylated sites (T175, S185, S195, S198, T205 and S208) were specifically identified, which might be strongly implicated in the aggregation properties of tau. Indeed, S208 has been reported to promote microtubule dysfunction and tau aggregation in MS analysis of PHF-tau purified from AD brains, in cultured cells as well as in transgenic mouse models of tauopathies and in postmortem brain samples of patients with AD and other tauopathies^38–40^. Also, S198, which was identified specifically in P2h, would also be related to tau aggregation since it has been reported as one of abnormal phosphorylation sites of PHF-tau^38^. T403 has also been identified in PHF-tau^38^. However, this phosphorylated site was identified in the S2h, P2h and P2 fractions in this study, suggesting that T403 might not affect tau aggregation. There are some controversies over whether the phosphorylation is a factor contributing to tau aggregation or if the phosphorylation occurs after tau aggregation^37^. Since many tau peptides in P2h were found to undergo phosphorylation, and most phosphorylation sites we identified in this study were included in LCRs (Fig. 5A), phosphorylation might strongly affect tau aggregation.

We further investigated the heat-stability and the aggregation properties of FUS and TDP-43 (Fig. 4). Since PTMs such as phosphorylation or ubiquitination have been reported to cause ALS^41,42^, we examined whether any PTMs were identified by LC–MS/MS. As a result, the FUS peptides oxidated at M321 and M464 residues were identified in the S2h heat-stable fraction, not in any other fractions. Accumulation of oxidized proteins, which is associated with a number of diseases including ALS and AD, reflects not only the rate of protein oxidation but also the rate of oxidized protein degradation^43^. Also, numerous studies have demonstrated the increased oxidative damage to protein in ALS by analyzing protein carbonyl levels which have been found to be elevated in ALS cases^44^. Therefore, the amount of oxidation at M321 and M464 residues might be implicated in the pathogenesis of ALS, as well as the heat-stability of FUS.

Since phosphorylation sites of TDP-43, including S379, S403/S404, S409 and S410, were identified in insoluble fractions of ALS by LC–MS/MS previously^45^, we assumed that the TDP-43 peptides phosphorylated at those sites would be identified in the P2h insoluble fraction. However, the phosphorylated peptides were not identified in any fractions. We then investigated whether the peptides containing those phosphorylation sites, but not being phosphorylated, were identified by LC–MS/MS. As a result, the identified peptides in this study were mostly located in the N-terminus of TDP-43, therefore, any peptides including those reported phosphorylation sites were not identified. Instead of phosphorylation, carbamidomethylation and carbamylation of TDP-43 were found in S2, P2 and P2h, which might be artificially introduced during sample preparation. Therefore, the effect of PTMs on the properties of TDP-43 could not be confirmed in this study.

In addition, Western blot showed some strong bands in the non-heat-treated insoluble P2 fraction of FUS (40 kDa and 65 kDa) and TDP-43 (43 kDa and 180 kDa) (Fig. 4). These bands might indicate that proteins in the S2 fraction could be insolubilized during centrifugation and shifted to P2. However, since 40 kDa and 65 kDa sized bands of FUS were detected not only in P2 but also in S2 and P2h, there is a possibility that these isoforms and fragments might undergo phase separation. The 180 kDa sized band of TDP-43 detected in P2 was stronger than those in S2 and P2h. This might indicate that the TDP-43 isoforms or fragments in certain size easily form polymers regardless of the heat-treatment.

IDPs containing LCRs can promote phase separation to form droplets in vitro, and thus are supposed to contribute to their compartmentalization in a regulated manner in vivo^46–48^. Therefore, the proteins with LCRs, which normally tend to form liquid-like phases, could end up taking more solid-like properties and forming toxic aggregates in diseased cases^49^. PTMs could be the most crucial regulation mechanism of phase separation. Phosphorylation is the most common PTM that an LCR can undergo, and there are many protein kinase dependent phosphorylation sites in LCRs^50,51^. These phosphorylated sites have divalent anions, therefore the solubility of a protein could increase. Also, those phosphorylated sites inhibit the interactions between molecules, therefore phosphorylation of LCRs could inhibit liquid phase formation. FUS and TDP-43 are the examples of IDPs that are related to human diseases and undergo liquid–liquid phase separation (LLPS)^41,46,52^. When a mutation occurs in the LCR of FUS, a formation of fibrils would be promoted by cross-β polymerization, which can be seen in prion-like domains. Therefore, the phase transition from liquid to hydrogel states in the mutant LCRs occurs more quickly than the wild-type LCRs^53^. Tau has also been reported to undergo LLPS under cellular conditions, and phase-separated tau droplets can serve as an intermediate toward tau aggregate formation^54^. An in vitro study demonstrated that disease-related mutation, P301L, and phospho-AT8 modifications enhanced LLPS of tau, which might be implicated in the pathological tau mechanisms^55^. Recently, this abnormal property of LLPS is thought to be an important mechanism of the pathogenesis of neurodegenerative diseases.

In order to investigate whether the heat-stable proteins identified in this study would undergo LLPS, we used a sequence-based prediction tool for LLPS proteins called PSPredictor (http://www.pkumdl.cn:8000/PSPredictor/) (Supplementary information 3). Tau (UniProt: P10637-2), for example, is predicted to be a potential phase separation protein (PSP) with a PSP score 0.9963. Among the proteins in the S2h, P2h, S2 and P2 datasets, 100 representative proteins containing LCRs from each dataset were selected and analyzed. Therefore, most of the selected heat-stable proteins in S2h included CBRs in their sequences. As a result, 84 proteins of S2h were predicted to be potential PSPs, while 15 proteins in P2h, 35 proteins in S2 and 38 proteins in P2 were predicted to be potential PSPs. In addition, the number of amyloid fibril regions in the proteins in S2h predicted by a computational aggregation prediction tool called PASTA2.0 (http://old.protein.bio.unipd.it/pasta2/index.html)^56^ was not significantly different from that of the proteins in other datasets. This might be because protein aggregation would be affected by many factors including PTMs, as suggested in this study, as well as by mutations such as expanded polyglutamine^32^. In fact, the number of predicted amyloid fibril regions of ⍺-syn (UniProt: O55042) is 20, and that of tau (UniProt: P10637-2) is 1. However, both proteins are well known to form aggregates. Although we could not find significant differences in the aggregation prediction results among the proteins of each dataset, we suggest that the heat-stable proteins identified in this study would be more likely to undergo LLPS compared to the proteins without heat-treatment.

Due to their amino acid composition biases, IDPs are characterized by the “turned out” response to changes in their environment. Under certain conditions, such as acidic pH, urea or high temperature^57–60^, a protein can lose its unique and ordered structure as well as its ability to function, and would then be considered denatured^61,62^. These features were used in the past to discover unfoldome, a portion of proteome which includes a set of IDPs^63^. In particular, the heat-treatment of mouse fibroblast cell extracts allowed for the identification of IDPs^58^, and large-scale proteomics revealed that the majority of heat-stable proteins isolated from mouse fibroblast cells were predicted to be substantially disordered^59^. In this study, we mainly discussed the heat-stable proteins in nervous systems directly prepared from mouse brains. Heat-stable proteins are not exactly the same as IDPs, but they share many features such as the presence of LCRs in their sequences. We performed an intrinsic disorder predisposition analysis of the proteins containing LCRs in all the datasets: S2h, P2h, S2 and P2. 100 representative proteins from each dataset were analyzed via computational disorder prediction tools, including PASTA2.0 and IUPred3 (https://iupred.elte.hu)^64^ (Supplementary information 3). Since the percentages of IDRs of ⍺-syn (UniProt: O55042) and tau (UniProt: P10637-2) are 32.85% and 60.98%, respectively, we counted the proteins containing more than 30% of IDRs in their sequences. As a result, 77 out of 100 proteins in S2h contained more than 30% of IDRs, while only 8 proteins in P2h contained more than 30% of IDRs. In S2 and P2, 22 and 26 proteins each contained more than 30% of IDRs. This indicates that many heat-stable proteins with LCRs and CBRs also contain IDRs as IDPs.

The heat-stable proteins in mouse nervous systems can be characterized by the abundance of CBRs. Although we could not account for the direct relationship between this feature and the aggregation properties of the heat-stable proteins, our analyses demonstrated that the presence of CBRs might be the crucial factor for a protein to undergo LLPS. Further research and analyses are necessary to investigate the pathological significance of heat-stable proteins and their implications for human diseases.

## Methods

### Purification of heat-stable proteins

This study is reported in accordance with ARRIVE guidelines (https://arriveguidelines.org). Animals were housed and treated in accordance with relevant guidelines and regulations set by Doshisha University, and all experimental procedures performed on mice were approved by the Doshisha University Animal Care and Use Committee. 22-week-old C57BL6J male mice were anesthetized and sacrificed with isoflurane. The mouse brains were taken and homogenized with a 1 ml homogenization buffer [50 mM PIPES, 0.5 mM MgCl_2_, 1 mM EGTA, 0.1 M EDTA, 0.5 M NaCl, 3 mM DTT] on ice at 900 rpm 20 times using a digital homogenizer (Iuchi, Tokyo, Japan). The brain homogenates were then moved to a 1.5 ml microcentrifuge tube and incubated on ice for 1 h, followed by centrifugation at 20,400×g for 30 min at 4 °C. The supernatant (S1) was collected and heated at 95 °C for 5 min (S1h), and both S1 and S1h fractions were centrifuged at 20,400×g for 30 min at 4 °C. The supernatant S2 and S2h were collected for further experiments. The pellets P2 and P2h were washed carefully with 500 µl of homogenization buffer twice to remove the remaining solution completely and were resuspended with 1 ml of homogenization buffer. The method was modified from that of Grundke-Iqbal et al.^17^. The protein concentrations were measured using the Micro BCA™ Protein Assay Kit (23,235, Thermo Scientific™).

### Western blot

Equal amounts of each brain fraction were loaded into a 5–20% SDS–polyacrylamide gel (e-PAGEL®, E-T520L, Atto), and the proteins were separated and transferred to polyvinylidene difluoride membranes (Immobilon®-P Transfer Membrane, Merck). The membranes were blocked with 2.5% goat serum in Tris Buffered Saline with Tween 20 (TBST) and incubated with the following antibodies at 4 °C overnight: anti-⍺-syn (D37A6, Cell Signaling Technology), anti-tau (T57120, BD Transduction), anti-MAP2 (M9942, Sigma-Aldrich), anti-phosphorylated-synuclein (pSyn #64, Wako), anti-phosphorylated-tau (PHF13 S396, 10-444, Cell Signaling Technology; Phospho-Tau S262, #44-750G, Invitrogen; AT8 S202/T205, 00-1566, Innogenetics), anti-FUS (HPA008784, Sigma-Aldrich; 11570-1-AP, Proteintech), and anti-TDP43 (G400, Cell Signaling Technology) antibodies in 2.5% goat serum/TBST (1:1000). The membranes were washed in TBST for 10 min twice and incubated in a horseradish peroxidase secondary antibody (Immobilon® Forte Western HRP Substrate) diluted with 2.5% goat serum/TBST (1:2000) for 1 h, at room temperature. The immunoreactive proteins were visualized via the application of the substrate for enhanced chemiluminescence (Luminata, Millipore). The signals were acquired using ImageQuant LAS-4000 (GE Healthcare).

### Protein digestion and LC–MS/MS

Protein tryptic digestion was performed with Filter-aided Sample Preparation (FASP) method^65^. Samples were mixed with UA solution (8 M Urea in 100 mM Tris–HCl, pH 8.0) in filter units (molecular weight cut-off 30 kDa, PT-1007, Aproscience) and centrifuged at 14,000×g for 15 min at room temperature, followed by the addition of IAA solution (0.05 M iodoacetamide in UA). The samples were then incubated for 20 min in the dark at room temperature and filtered by centrifuging them at 14,000×g for 10 min. After the centrifugation, 100 µl of UA was added to the filter, followed by centrifugation at 14,000×g for 15 min. This step was repeated twice. The proteins trapped on the filters were washed with AmBic solution (50 mM ammonium bicarbonate in Milli-Q) three times, and the residual proteins were enzymatically digested by 40 µl of 0.2 μg/μl modified trypsin (V511A, Promega) in AmBic solution (enzyme to protein ratio 1: 100) overnight at 37 °C. The digested proteins were collected by centrifuging at 14,000×g for 10 min after adding a total of 80 µl AmBic solution and 50 µl of 0.5 M NaCl to the filter units. The filtrates were then applied to the liquid-chromatography mass spectrometry (LC–MS/MS) system.

In-gel digestion was performed using the Thermo Scientific™ In-Gel Tryptic Digestion Kit (89,871, ThermoScientific) in order to obtain and identify peptides of interest stained by Coomassie Brilliant Blue (CBB). The digested proteins were analyzed by LC–MS/MS.

To obtain MS/MS spectrum data of the peptides, the digested peptides were separated by EASY-nLC 1000 (Thermo Fisher Scientific) and ionized with nano-ESI, followed by analyzation using a QExactive hybrid quadrupole-orbitrap mass spectrometer (Thermo Fisher Scientific). Based on this peptide information, proteins were identified using Proteome Discoverer version 2.2 (PD2.2, Thermo Fisher Scientific) with the MASCOT search engine software (Matrix Science)^66^.

### Bioinformatic analyses

DAVID Bioinformatics Resources 6.8 (https://david.ncifcrf.gov) was used to investigate the sequence features of the proteins obtained by LC–MS/MS. For bioinformatic analysis, Bonferroni adjusted *p* < 0.05 was considered significant. A platform of tools for low complexity called PlaToLoCo (http://platoloco.aei.polsl.pl/#!/query)^29^ was used to annotate low complexity regions in the proteins obtained by LC–MS/MS. We used fLPS-strict tool which focuses on searching for compositionally biased regions and identifies roughly the same proportion of compositionally biased segments in all the genomes. PSPredictor (http://www.pkumdl.cn:8000/PSPredictor/)^67^ was used to predict potential phase separation proteins. Proteins with a PSP score of > 0.5 were considered to be potential phase separation proteins. The computational disorder prediction tools called PASTA2.0 (http://old.protein.bio.unipd.it/pasta2/index.html)^56^ and IUPred3 (https://iupred.elte.hu)^64^ were used for an intrinsic disorder predisposition analysis. The proteins containing more than 30% of IDRs were counted and analyzed.

## Supplementary Information

Supplementary Information 1.

Supplementary Information 2.

Supplementary Information 3.

Supplementary Information 4.
