# Supplementary material for: Proteomic analysis of heat-stable proteins revealed an increased proportion of proteins with compositionally biased regions
Source: Sci Rep. 2022 Mar 14;12:4347. doi: 10.1038/s41598-022-08044-z (PMC8921518; doi:10.1038/s41598-022-08044-z)
Supplement: Supplementary file 4 — Supplementary Information 4. [file 41598_2022_8044_MOESM4_ESM.pdf]

Supplementary Information

Proteomic analysis of heat-stable proteins revealed an increased proportion of proteins with compositionally biased regions

Hongsun Park, Tomoyuki Yamanaka, Nobuyuki Nukina

Separate excel files

Supplementary dataset file 1

Supplementary dataset file 2

Supplementary dataset file 3

Supplementary Tables

Table S1. The sequence features of P2 proteins analyzed by DAVID.

	Term	Count	%	List Total	Bonferroni
1	nucleotide phosphate-binding region: GTP	126	3.77132595	3243	1.21E-15
2	transit peptide: Mitochondrion	168	5.0284346	3243	6.44E-14
3	binding site: Substrate	112	3.35228973	3243	6.60E-11
4	repeat: HEAT 3	29	0.86800359	3243	3.38E-08
5	repeat: HEAT 4	26	0.77821012	3243	7.38E-07
6	repeat: HEAT 5	23	0.68841664	3243	1.03E-06
7	short sequence motif: Effector region	46	1.37683328	3243	2.86E-06
8	metal ion-binding site: Magnesium	49	1.46662676	3243	4.03E-06
9	domain: PCI	17	0.50882969	3243	4.64E-06
10	repeat: HEAT 2	29	0.86800359	3243	6.66E-06
11	repeat: HEAT 1	29	0.86800359	3243	6.66E-06
12	nucleotide phosphate-binding region: ATP	247	7.39299611	3243	9.07E-06
13	lipid moiety-binding region: S-geranylgeranyl cysteine	48	1.4366956	3243	9.85E-06
14	repeat: WD 5	75	2.24483688	3243	1.49E-05
15	repeat: WD 4	79	2.36456151	3243	3.17E-05
16	repeat: WD 3	82	2.45435498	3243	3.97E-05
17	repeat: WD 2	82	2.45435498	3243	1.12E-04
18	repeat: WD 1	82	2.45435498	3243	1.12E-04
19	region of interest: Calmodulin-binding	27	0.80814128	3243	2.16E-04
20	repeat: HEAT 6	19	0.56869201	3243	2.36E-04
21	domain: PH	71	2.12511224	3243	2.74E-04
22	repeat: WD 6	60	1.7958695	3243	4.57E-04
23	splice variant	966	28.913499	3243	5.17E-04
24	domain: CH 2	16	0.47889853	3243	8.37E-04
25	domain: CH 1	16	0.47889853	3243	8.37E-04
26	region of interest:Substrate binding	51	1.52648908	3243	0.00184942
27	nucleotide phosphate-binding region: NADP	30	0.89793475	3243	0.00198894
28	domain: SH3	58	1.73600718	3243	0.00227733
29	binding site: NADP	17	0.50882969	3243	0.00323605
30	domain: RRM 3	25	0.74827896	3243	0.0081306
31	repeat: HEAT 7	15	0.44896738	3243	0.01903594
32	active site: Proton acceptor	176	5.26788387	3243	0.02575644
33	repeat: WD 7	45	1.34690213	3243	0.03637115
34	domain: C2 2	26	0.77821012	3243	0.03781453
35	domain: C2 1	26	0.77821012	3243	0.03781453
36	domain: RRM 2	45	1.34690213	3243	0.04564407
37	domain: RRM 1	45	1.34690213	3243	0.04564407

Table S2. The sequence features of P2h proteins analyzed by DAVID.

	Term	Count	%	List Total	Bonferroni
1	transit peptide: Mitochondrion	180	5.75079872	3030	3.15E-22
2	nucleotide phosphate-binding region: GTP	126	4.02555911	3030	3.13E-18
3	short sequence motif: Effector region	50	1.59744409	3030	6.31E-10
4	lipid moiety-binding region: S-geranylgeranyl cysteine	53	1.69329073	3030	7.02E-10
5	binding site: Substrate	94	3.00319489	3030	1.08E-05
6	domain: PCI	16	0.51118211	3030	3.00E-05
7	nucleotide phosphate-binding region: ATP	229	7.31629393	3030	8.66E-05
8	nucleotide phosphate-binding region: NADP	31	0.99041534	3030	8.94E-05
9	topological domain: Mitochondrial intermembrane	28	0.89456869	3030	3.39E-04
10	metal ion-binding site: Magnesium	43	1.37380192	3030	5.76E-04
11	repeat: HEAT 5	18	0.57507987	3030	0.01027529
12	repeat: HEAT 4	20	0.63897764	3030	0.01596039
13	active site: Proton acceptor	167	5.33546326	3030	0.01878577
14	repeat: HEAT 3	21	0.67092652	3030	0.02288084
15	region of interest: Calmodulin-binding	23	0.73482428	3030	0.02559034
16	calcium-binding region: 1	43	1.37380192	3030	0.02826959
17	calcium-binding region: 2	40	1.27795527	3030	0.03020487
18	topological domain: Vesicular	20	0.63897764	3030	0.04154398

Table S3. The sequence features of S2 proteins, with and without LCRs, analyzed by DAVID.

**S2**	
LCR+	LCR-
2,955 (84.2%)	553
nucleotide phosphate-binding region: ATP	binding site: Substrate
transit peptide: Mitochondrion	lipid moiety-binding region: S-geranylgeranyl cysteine
splice variant	nucleotide phosphate-binding region: GTP
domain: SH3	short sequence motif: Effector region
repeat: HEAT 3	transit peptide: Mitochondrion
nucleotide phosphate-binding region: GTP	region of interest: Substrate binding
domain: PH	metal ion-binding site: Magnesium
repeat: HEAT 4	nucleotide phosphate-binding region: NADP
repeat: WD 5	propeptide: Removed in mature form
repeat: HEAT 2	active site: Proton donor
repeat: HEAT 1	
repeat: WD 6	
repeat: HEAT 5	
repeat: WD 3	
domain: C2	
repeat: WD 4	
repeat: WD 7	
binding site: ATP	
compositionally biased region: Glu-rich	
repeat: WD 2	
repeat: WD 1	
metal ion-binding site: Magnesium	
domain: Guanylate kinase-like	
region of interest: Calmodulin-binding	

* See supplementary dataset file 1 for each protein list.

Table S4. The proportion of proteins with CBRs in S2h and S2, with the presence of LCRs.

**S2h (LCR+)**		**S2 (LCR+)**	
Sequence features	# Proteins	Sequence features	# Proteins
compositionally biased region: Pro-rich	105	compositionally biased region: Glu-rich	72
compositionally biased region: Glu-rich	47		
compositionally biased region: Lys-rich	29		
compositionally biased region: Ser-rich	59		
compositionally biased region: Arg/Ser-rich (RS domain)	11		
compositionally biased region: Poly-Glu	52		
domain:P compositionally biased region: Gly-rich	34		
# Total S2h CBR+ proteins	337	# Total S2 CBR+ proteins	72
# Analyzed S2h LCR+ proteins	1156	# Analyzed S2 LCR+ proteins	2846
%	29.2	%	2.6

* See supplementary dataset file 1 for each protein list.

Table S5. The sequence features of P2 proteins, with and without LCRs, analyzed by DAVID.

**P2**	
LCR+	LCR-
2,807 (84.0%)	536
splice variant	binding site: Substrate
repeat: HEAT 3	lipid moiety-binding region: S-geranylgeranyl cysteine
domain: PH	nucleotide phosphate-binding region: GTP
nucleotide phosphate-binding region: ATP	short sequence motif: Effector region
repeat: HEAT 4	region of interest: Substrate binding
repeat: HEAT 5	transit peptide: Mitochondrion
repeat: HEAT 1	metal ion-binding site: Magnesium
repeat: HEAT 2	nucleotide phosphate-binding region: NADP
transit peptide: Mitochondrion	active site: Proton donor
domain: SH3	
repeat: HEAT 6	
region of interest: Calmodulin-binding	
repeat: WD 5	
repeat: WD 3	
repeat: WD 4	
nucleotide phosphate-binding region: GTP	
repeat: WD 1	
repeat: WD 2	
domain: C2 1	
domain: C2 2	
repeat: WD 6	
domain: C2	
domain: RRM 1	
domain: RRM 2	
domain: RRM 3	
domain: CH 2	
domain: CH 1	
repeat: WD 7	
repeat: HEAT 7	
domain: AGC-kinase C-terminal	

* See supplementary dataset file 2 for each protein list.

Table S6. The sequence features of P2h proteins, with and without LCRs, analyzed by DAVID.

**P2h**	
LCR+	LCR-
2,646 (84.5%)	486
transit peptide: Mitochondrion	lipid moiety-binding region: S-geranylgeranyl cysteine
nucleotide phosphate-binding region: ATP	binding site: Substrate
splice variant	short sequence motif: Effector region
nucleotide phosphate-binding region: GTP	nucleotide phosphate-binding region: GTP
repeat: HEAT 5	transit peptide: Mitochondrion
repeat: HEAT 4	nucleotide phosphate-binding region: NADP
repeat: HEAT 3	region of interest: Substrate binding
domain: PH	active site: Proton donor
topological domain: Vesicular	propeptide: Removed in mature form
repeat:HEAT 1	binding site: NADP
region of interest: Calmodulin-binding	
repeat:HEAT 2	
transmembrane region	
domain:C2	

* See supplementary dataset file 2 for each protein list.

Table S7. The sequence features of the shared proteins between S2 and S2h analyzed by DAVID.

	Term	Count	%	List Total	Bonferroni
1	repeat:4	21	2.7	766	0.00125612
2	calcium-binding region:1	20	2.5	766	0.00370967
3	domain:EF-hand 1	23	2.9	766	0.01161888
4	calcium-binding region:2	18	2.3	766	0.01517009
5	repeat:8	13	1.6	766	0.01965488
6	domain:EF-hand 2	22	2.8	766	0.03542429
7	transit peptide:Mitochondrion	43	5.5	766	0.04193638
8	repeat:2	24	3.0	766	0.04383682
9	compositionally biased region:Glu-rich	28	3.6	766	0.04845282

* See supplementary dataset file 1 for the protein list.

Table S8. The sequence features of S2 proteins analyzed by DAVID.
(The shared proteins between S2 and S2h were excluded.)

	Term	Count	%	List Total	Bonferroni
1	transit peptide:Mitochondrion	158	5.8	2629	4.11E-19
2	nucleotide phosphate-binding region:ATP	248	9.1	2629	4.45E-17
3	nucleotide phosphate-binding region:GTP	110	4.0	2629	3.08E-15
4	short sequence motif:Effector region	47	1.7	2629	2.79E-10
5	lipid moiety-binding region:S-geranylgeranyl cysteine	49	1.8	2629	9.47E-10
6	metal ion-binding site:Magnesium	46	1.7	2629	1.17E-07
7	binding site:Substrate	87	3.2	2629	2.12E-06
8	repeat:WD 5	64	2.4	2629	4.88E-05
9	active site:Proton acceptor	160	5.9	2629	5.05E-05
10	repeat:WD 3	70	2.6	2629	9.54E-05
11	repeat:WD 4	67	2.5	2629	1.24E-04
12	repeat:WD 6	53	1.9	2629	2.06E-04
13	repeat:WD 2	70	2.6	2629	2.32E-04
14	repeat:WD 1	70	2.6	2629	2.32E-04
15	nucleotide phosphate-binding region:NADP	28	1.0	2629	2.89E-04
16	binding site:ATP	133	4.9	2629	4.82E-04
17	domain:PCI	14	0.5	2629	8.13E-04
18	domain:Guanylate kinase-like	15	0.6	2629	0.00103648
19	binding site:NADP	16	0.6	2629	0.00114384
20	region of interest:Substrate binding	44	1.6	2629	0.00294306
21	nucleotide phosphate-binding region:FAD	25	0.9	2629	0.005011
22	repeat:WD 7	40	1.5	2629	0.01445704

Supplementary Figures

We adjusted the contrast and the brightness of the original Western blot results analyzed by ImageQuant LAS-4000 (GE Healthcare) using ImageJ because saving the images as TIF files caused them to brighten. To show an example of this, we have included Figure S1, the MAP2 original data, the version saved as a TIF, and the edited version. In figure S2, S3 and S4, we have included the full-sized images of the results from CBB staining and Western blot that have not been cropped nor processed.

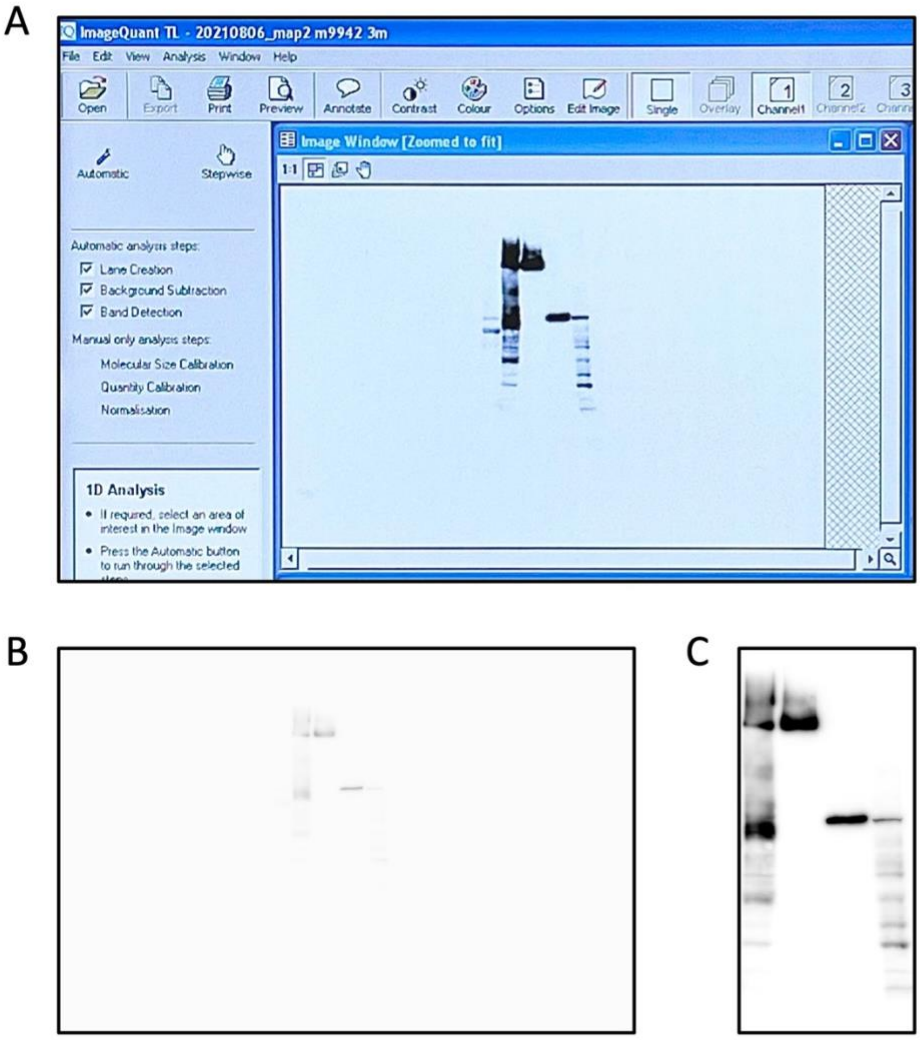

Figure S1. An example of a Western blot result of each fraction stained with the antibodies to MAP2 to compare the original data, the version saved as a TIF and the edited version.

(A) The original data analyzed by ImageQuant LAS-4000 (GE Healthcare). **(B)** The image saved as a TIF file. **(C)** The edited version (Figure 1C).

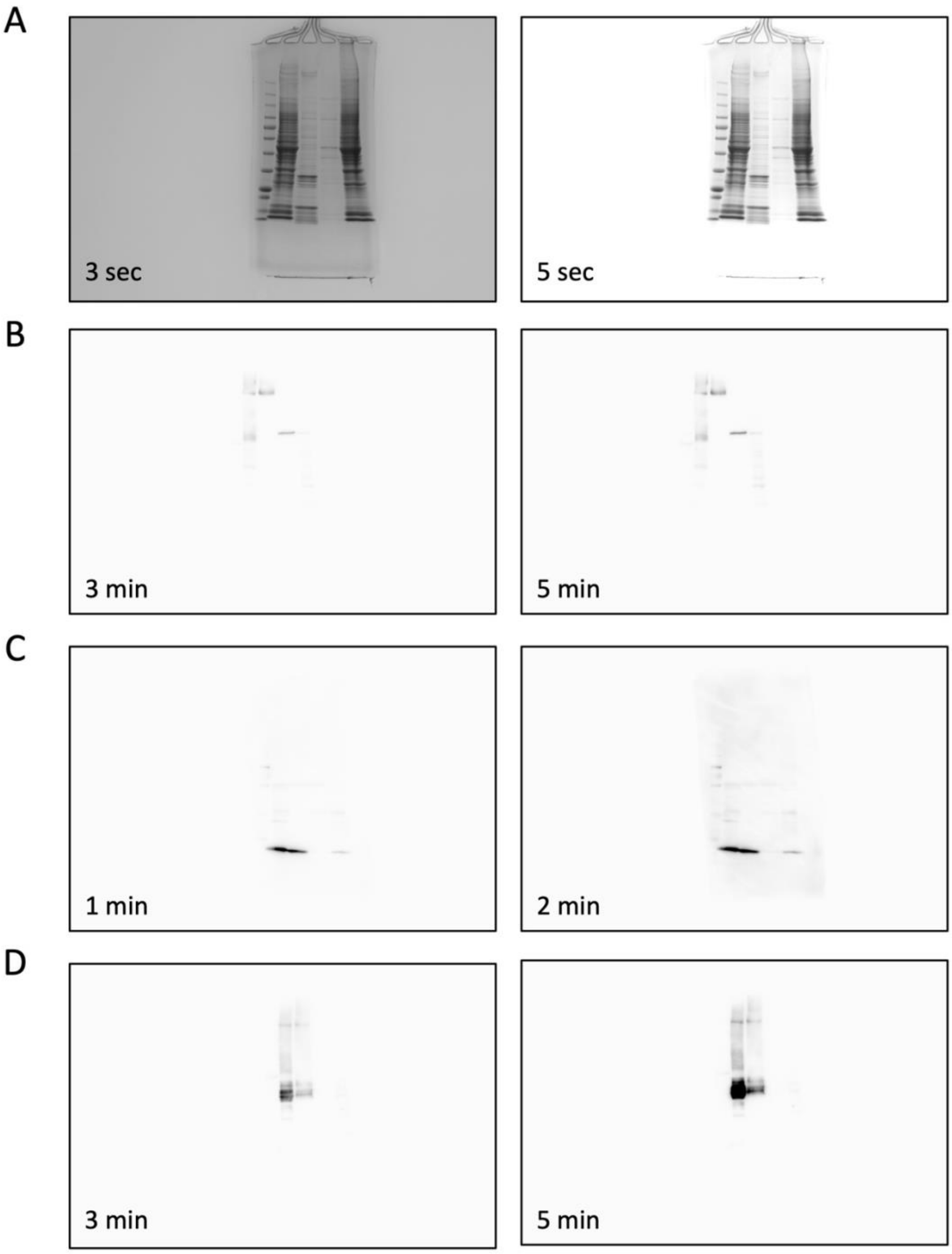

Figure S2. The full-sized images of CBB staining and Western blot results in Figure 1. **(A)** The CBB staining result of each fraction. The Western blot results of each fraction stained with the antibodies to MAP2 **(B)**, α-syn **(C)** and tau **(D)**. These images were cropped in order to remove the protein marker lanes and the white spaces that are irrelevant to the results. The exposure times are shown in each image.

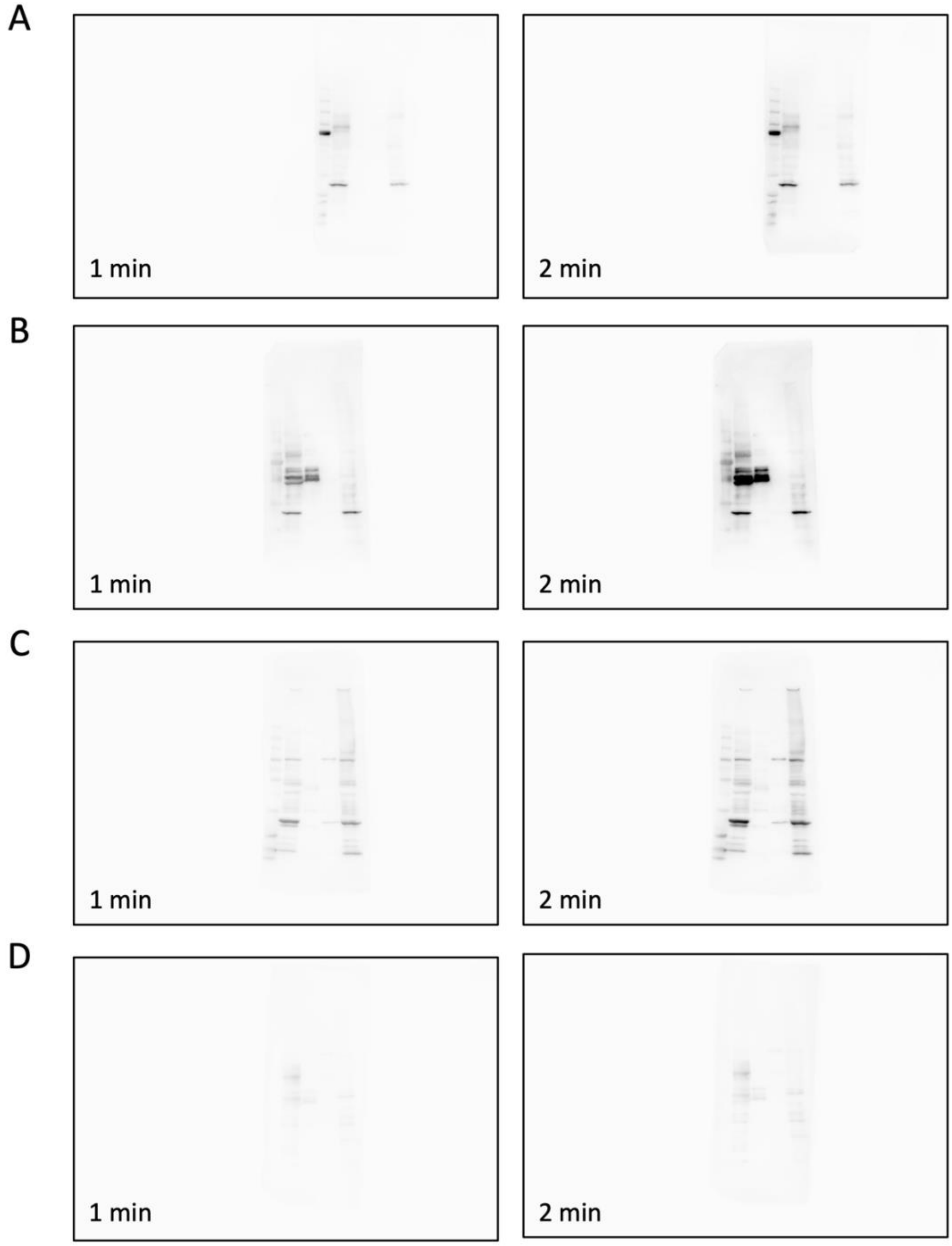

Figure S3. The full-sized images of Western blot results in Figure 3.

The Western blot results of each fraction stained with the antibodies to p-syn **(A)**, p-tau S396 **(B)**, p-tau S262 **(C)** and p-tau S202/T205 (AT8) **(D)**. These images were cropped in order to remove the protein marker lanes and the white spaces that are irrelevant to the results. The exposure times are shown in each image.

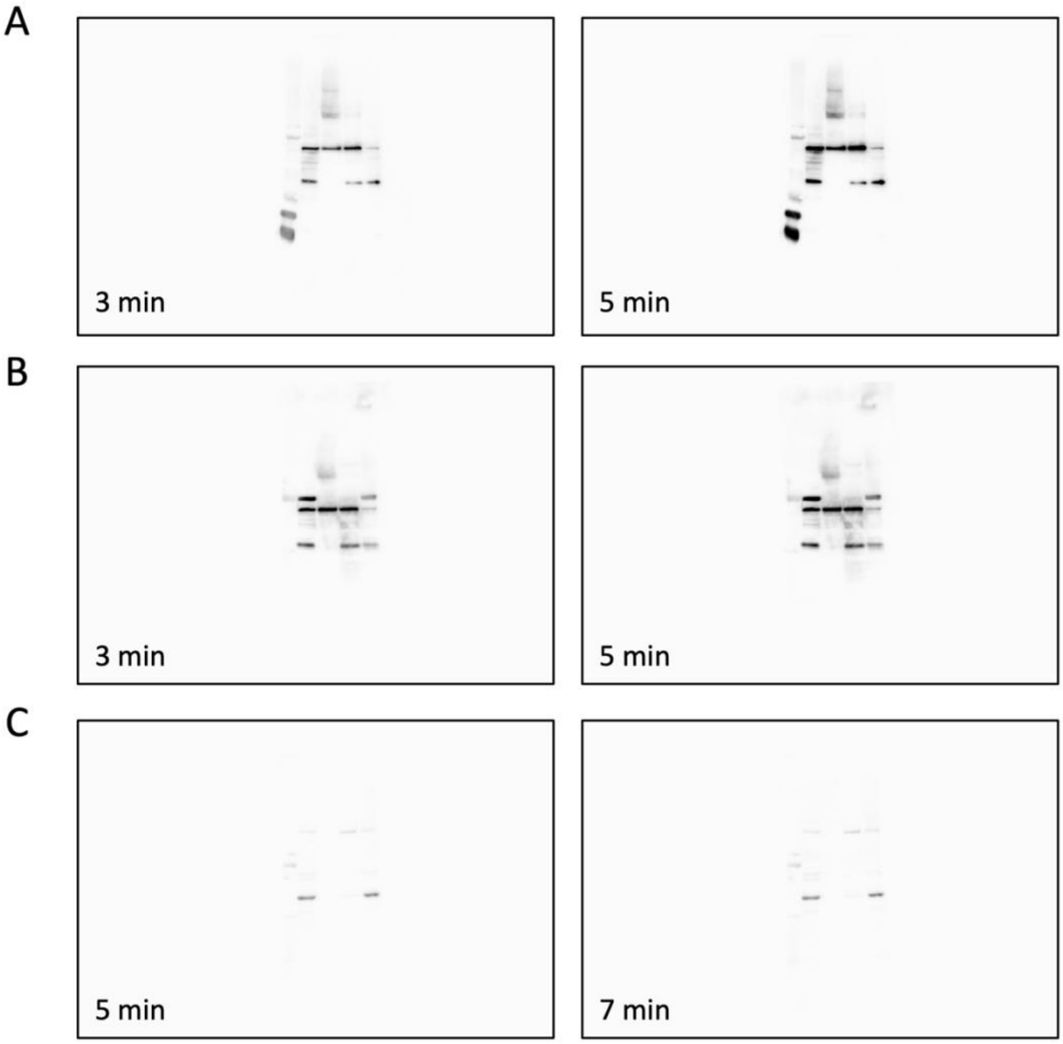

Figure S4. The full-sized images of Western blot results in Figure 4.

The Western blot results of each fraction stained with the antibodies to FUS (Proteintech, 11570-1-AP) **(A)**, FUS (Sigma-Aldrich, HPA008784) **(B)** and TDP-43 **(C)**. These images were cropped in order to remove the protein marker lanes and the white spaces that are irrelevant to the results. The exposure times are shown in each image.
